# Commentary: Meta-Analyses Reporting the Prognostic Value of Androgen Receptor Splice Variant 7 in Castration-Resistant Prostate Cancer

**DOI:** 10.3389/fonc.2020.602992

**Published:** 2020-11-30

**Authors:** Michael W. Kattan, Ronald M. Bukowski

**Affiliations:** ^1^ Department of Quantitative Health Sciences in the Lerner Research Institute, Cleveland Clinic, Cleveland, OH, United States; ^2^ Taussig Cancer Center, Cleveland Clinic, Cleveland, OH, United States

**Keywords:** prostate cancer, randrogen receptor, splice variant 7, castration-resistant prostate cancer (CRPC), prognostication

## Introduction

The concurrent systematic reviews recently published in *Frontiers in Oncology* by Zhize Wang et al. (1) and Jiaxin Wang et al. (2) provide a very similar report on the prognostic value of the androgen receptor splice variant 7 (AR-V7) in castration-resistant prostate cancer. As these might appear, at first glance, to be nearly identical, we believed it was pertinent to herein highlight the differences and summarize the findings.

### Selection Criteria for the Meta-Analyses

The paper by Z. Wang examined studies published on PubMed, Embase, and the Web of Science from inception to February 2020, while J. Wang restricted their search to PubMed, Embase, and MEDLINE from January 1974 to September 2019. More importantly, the paper by J. Wang studied *metastatic* castration-resistant prostate cancer, while Z. Wang et al. looked only at castration-resistant prostate cancer. In addition, if patients were not receiving chemotherapy, J. Wang required patients to be treated by androgen receptor signaling inhibitors (ARSIs) or chemotherapy, while Z. Wang included only results from patients treated with novel hormonal therapy (NHT). For these reasons (**Table 1**), and perhaps others, the numbers of studies included for analysis differed: 21 in the J. Wang study and 36 in the Z. Wang study. Having said that, notable overlap exists between the studies included by each.

**Table 1:** Characteristics of the Systematic Reviews by Z. Wang et al. and J. Wang et al.

Sources	Zhize Wang et al.	Jiaxin Wang et al.
	PubMed, Embase, Web of Science	PubMed, Embase, MEDLINE
*Publication period considered*	Inception to February 2020	January 1974 to September 2019
*Inclusion criteria*	1) Studies reporting on CRPC and AR-V7; 2) Results expressed as an 86% positive rate in AR-V7 before and after treatment in CRPC. Other included results were the PSA response rate, PFS, or OS after NHT or chemotherapy; 3) Results reported from clinical trials including RCTs and non-randomized studies; 4) Studies in English or with a translation provided if published in any other language.	1) Studies reporting on the association between AR-V7 status at baseline and time-to-events outcomes for mCRPC patients treated with ARSis or chemotherapy, including PSA response, clinical and/or radiographic PFS or OS; 2) Odds ratios (ORs) or hazard ratios (HRs) with corresponding 95% confidence intervals (CIs) were reported directly or could be calculated; 3) Clinical studies performed with adults and were published in English.
*Exclusion criteria*	1) Case Reports, Comments, Editorials, Letters, or Reviews; 2) Treatment was neither novel hormonal therapy nor chemotherapy, or not clearly mentioned; 3) Studies reported only the AR-V7-positive proportion before or after treatment in CRPC patients; 4) Studies lacking results of therapy response rate, PFS, or OS; 4) Studies involving non-human subjects.	1) Reviews, Case Reports, Comment, Editorials, or Meta-Analysis; 2) Studies containing only an AR-V7-positive or an AR-V7-negative cohort but not both; 3) Studies reporting only the Kaplan–Meier survival curve without available HRs and 95% CI.

## Statistical Analysis

Both of these PROSPERO-registered systematic reviews used nearly identical and customary, meta-analytic approaches. While different software packages were used, this should hopefully have no impact on the results. Z. Wang decided whether to use fixed *vs.* random effects models based on the I^2^ value, while J. Wang used exclusively random effects models. Both models have pros and cons (3).

## Reporting of Results

Both studies looked at overall survival, progression-free survival, and prostate specific antigen (PSA) response after chemotherapy. However, Z. Wang also looked at AR-V7 positivity before and after treatment, while J. Wang furthermore subdivided results by method of detection.

## Findings

Not surprising—though reassuringly, given the overlap between the two meta-analysis—the findings between the two studies were quite similar: AR-V7-positive patients have worse prognosis, a trend that held regardless of treatment and endpoint. The hazard ratios for progression-free survival and overall survival were fairly similar (again, unsurprisingly, considering the included studies overlapped considerably). More difficult to reconcile were the odds ratios for PSA response. Z. Wang concluded that AR-V7-positive patients had a decreased PSA response rate (OR 0.13 for NHT, 0.63 for chemotherapy). J. Wang concluded AR-V7-positive patients had lower response rates (OR 6.01 for ARSIs, 2.23 for chemotherapy). Looking at the OR values, this would be a higher odds of response, rather than a lower, for the J. Wang study. It could be that J. Wang calculated the OR for AR-V7 negative, instead of positive, or that they calculated the OR for a non-response, rather than a PSA response. The fix to either is taking the reciprocal, which produces ORs of 0.17 and 0.45, respectively, somewhat in line with those of the Z. Wang study.

## Conclusions

Both studies argue for a prognostic role of AR-V7, with positive patients faring worse. The major limitation of studies like these is that they are not multivariable in nature. There are several prediction models available for men with progressive or hormone-refractory metastatic prostate cancer (4, 5). They use a variety of predictors and have reasonable accuracy. Thus, from the patient counseling perspective, one might want to know if AR-V7 positivity adds incremental value as a predictor, as this is the critical hurdle for any new biomarker (6). This is an important future research question.

## Author Contributions

MK wrote this article. RB reviewed and advised on this piece. All authors contributed to the article and approved the submitted version.

## Funding

Code DSC-08001617819PRD.

## Conflict of Interest

The authors declare that the research was conducted in the absence of any commercial or financial relationships that could be construed as a potential conflict of interest.
